# Supplementary material for: A secret from a hidden world: A new glassfrog of the genus Nymphargus (Anura: Centrolenidae) from Cordillera del Cóndor, Ecuador
Source: PLoS One. 2026 Apr 8;21(4):e0345097. doi: 10.1371/journal.pone.0345097 (PMC13061190; doi:10.1371/journal.pone.0345097)
Supplement: S1 Table — (PDF) [file pone.0345097.s001.pdf]

S1 Table. Primers used in this study.

Gene	FPrimer	RPrimer	Reference
12S	tPhe-frog	tVal-frog	[65]
ND-1	ND1-210F	WL384	[66]
	WL379b	tMet-frog	[65,67]
	ND1-F1	WL384	[66]
C-MYC	cmyc1U	cmyc-ex2 R	[65,68]
