# Supplementary figures and images for: A secret from a hidden world: A new glassfrog of the genus Nymphargus (Anura: Centrolenidae) from Cordillera del Cóndor, Ecuador
Source: PLoS One. 2026 Apr 8;21(4):e0345097. doi: 10.1371/journal.pone.0345097 (PMC13061190; doi:10.1371/journal.pone.0345097)

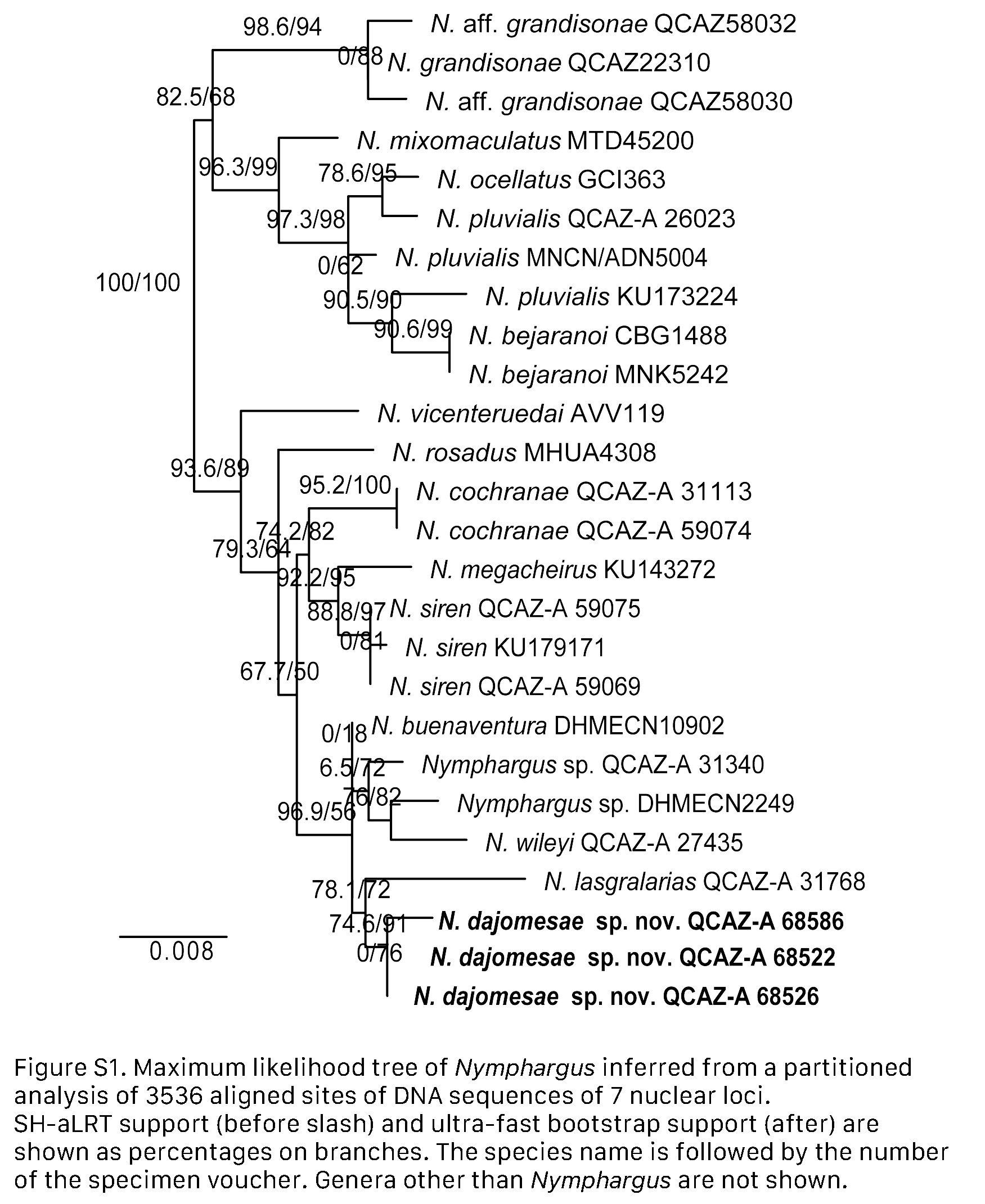

Supplement: S1 Fig — SH-aLRT support (before slash) and ultra-fast bootstrap support (after) are shown as percentages on branches. The species name is followed by the number of the specimen voucher. Genera other than Nymphargus are not shown. (TIF) [file pone.0345097.s002.tif]
