# Supplementary material for: A secret from a hidden world: A new glassfrog of the genus Nymphargus (Anura: Centrolenidae) from Cordillera del Cóndor, Ecuador
Source: PLoS One. 2026 Apr 8;21(4):e0345097. doi: 10.1371/journal.pone.0345097 (PMC13061190; doi:10.1371/journal.pone.0345097)
Supplement: S1 Appendix — (PDF) [file pone.0345097.s003.pdf]

S1 Appendix: Specimens examined

Nymphargus anomalus: ECUADOR: NAPO: KU 143299 (Holotype), QCAZ 41313. PASTAZA: QCAZ 45706 – 9, 49623. TUNGURAHUA: QCAZ 63380.

Nymphargus cariticommatus: ECUADOR: MORONA-SANTIAGO: KU 202805 (Paratype), USNM 288435-6. ZAMORA CHINCHIPE: QCAZ 33977.

Nymphargus chami: COLOMBIA: RISARALDA: ICN 32079 (Holotype). ANTIOQUIA: ICN 8666 (Paratype), ICN 10640 (Paratype).

Nymphargus cochranae: ECUADOR: NAPO: USNM 284304-6, 286632-36, USNM 286638. QCAZ 79096 – 99. ORELLANA: USNM 288452. ZAMORA-CHINCHIPE: DFCH D100-1, FHGO 2804.

Nymphargus colomai: ECUADOR: ZAMORA CHINCHIPE: QCAZ 41591, 41592, 41641 (Paratypes).

Nymphargus cristinae: COLOMBIA: ANTIOQUIA: ICN 18645 (Holotype), ICN 18643-4 (Paratype), 18646-9 (Paratype).

Nymphargus griffithsi: ECUADOR: PICHINCHA: USNM 286659, USNM 286662-64, 286667-77. CARCHI: QCAZ 39992, 39994, 12572. IMBABURA: QCAZ 31767- 69.

Nymphargus laurae: ECUADOR: ORELLANA: USNM 288453 (Holotype).

Nymphargus mariae: PERU: HUANUCO: KU 174713 (H, *C. mariae*). ECUADOR: PASTAZA: MCZ 91187 (H, *C. puyoensis*), USNM 291298. NAPO: DFCH-USFQ D285. ORELLANA: QCAZ 7104, 7499.

Nymphargus posadae: COLOMBIA: CAUCA: ICN 11307 (H), ICN 7447-50 (P). ECUADOR: SUCUMBIOS: USNM 288464-5.

Nymphargus prasinus: COLOMBIA: VALLE DEL CAUCA: KU 169693 (H), KU 169691-92 (P).

Nymphargus sucre: ECUADOR: MORONA SANTIAGO: QCAZ 68099 – 103.

Nymphargus sp.: ECUADOR: ZAMORA-CHINCHIPE: DHMECN 1974, 2249.

Nymphargus wileyi: ECUADOR: NAPO: QCAZ 22367 – 69, 22389, 26057, 32725.
