# Supplementary material for: A secret from a hidden world: A new glassfrog of the genus Nymphargus (Anura: Centrolenidae) from Cordillera del Cóndor, Ecuador
Source: PLoS One. 2026 Apr 8;21(4):e0345097. doi: 10.1371/journal.pone.0345097 (PMC13061190; doi:10.1371/journal.pone.0345097)
Supplement: Attachment — Submitted filename: Response_to_reviewers_auresp_2.docx [file pone.0345097.s007.docx]

RESPONSE TO REVIEWERS:

- “This sounds odd. Wouldn't it make more sense to just mention that all authors contributed equally?”

We thank the reviewer for this comment. We have followed his/her recommendation.

- “Please replace the current Spanish text within the figure with "Type locality"”

The requested change was made. The new version of the Figure is being uploaded.

- “I count six in the list you provide, please correct where necessary”
  Thanks for noticing this error. It has been fixed.
- “Any temperature data?”
  Unfortunately, temperature data is unavailable. We added a statement with that clarification in the methodology section.
